# The Russians Are the Fastest in Marathon Cross-Country Skiing: The “Engadin Ski Marathon”

**DOI:** 10.1155/2017/9821757

**Published:** 2017-08-21

**Authors:** Pantelis Theodoros Nikolaidis, Jan Heller, Beat Knechtle

**Affiliations:** ^1^Exercise Physiology Laboratory, Nikaia, Greece; ^2^Faculty of Health Sciences, Metropolitan College, Athens, Greece; ^3^Faculty of Physical Education and Sport, Charles University, Prague, Czech Republic; ^4^Gesundheitszentrum St. Gallen, St. Gallen, Switzerland; ^5^Institute of Primary Care, University of Zurich, Zurich, Switzerland

## Abstract

It is well known that athletes from a specific region or country are dominating certain sports disciplines such as marathon running or Ironman triathlon; however, little relevant information exists on cross-country skiing. Therefore, the aim of the present study was to investigate the aspect of region and nationality in one of the largest cross-country skiing marathons in Europe, the “Engadin Ski Marathon.” All athletes (*n* = 197,125) who finished the “Engadin Ski Marathon” between 1998 and 2016 were considered. More than two-thirds of the finishers (72.5% in women and 69.6% in men) were Swiss skiers, followed by German, Italian, and French athletes in both sexes. Most of the Swiss finishers were from Canton of Zurich (20.5%), Grisons (19.2%), and Berne (10.3%). Regarding performance, the Russians were the fastest and the British the slowest. Considering local athletes, finishers from Canton of Uri and Glarus were the fastest and those from Canton of Geneva and Basel the slowest. Based on the findings of the present study, it was concluded that local athletes were not the fastest in the “Engadin Ski Marathon.” Future studies need to investigate other cross-country skiing races in order to find the nationalities and regions of the fastest cross-country skiers.

## 1. Introduction

It is well known that athletes from a specific region or country are dominating certain sports disciplines. For example, runners from East Africa (i.e., Kenia and Ethiopia) are the fastest in track and road running up to the marathon distance [1, 2]. It has been shown that the fastest runners from Kenya and Ethiopia come from very specific regions. In Kenya, most national and international athletes come from the Rift Valley province and belong to the Kalenjin ethnic group and Nandi subtribe [3]. In Ethiopia, most marathon runners are from the regions of Arsi and Shewa [4].

For other sports disciplines such as triathlon, an effect of nationality on performance has also been observed: for example, European athletes dominate performances in Double Iron ultra-triathlons [5]. Furthermore, it has been shown that preferably local athletes are dominating specific races of various endurance sports. For example, the fastest triathletes competing in “Norseman Xtreme Triathlon” held in Norway were Norwegian women and men [6] and the fastest triathletes competing in “Ironman Hawaii” held in Hawaii, USA, were US-American women and men [7]. Greek men were the faster in “Spartathlon” (246 km) held in Europe, whereas American ultra-marathoners dominated both participation and performance in “Badwater” (217 km) in the USA [8]. For swimmers crossing the English Channel between England and France, most of the competitors were from Great Britain, USA, Australia, and Ireland. Although most swimmers were from England, British swimmers were, however, not the fastest. The fastest female swimmers were from the USA, Australia, and Great Britain and the fastest male swimmers were from the USA, Great Britain, and Australia [9, 10].

Cross-country skiing is another sport discipline where athletes from a specific origin might dominate since this sport discipline can only be held in regions with snow and cold [11]. Following the FIS (Fédération Internationale de Ski, http://www.fis-ski.com/), the best athletes in the Cross-Country World Cup are from Scandinavian countries such as Norway, Sweden, and Finland, but also from Russia, the USA, and countries from the region of the Alps in Europe (http://www.fis-ski.com/cross-country/).

In Switzerland, the “Engadin Ski Marathon” is held since 1969 and is one of the largest cross-country ski marathons in Europe (http://www.engadin-skimarathon.ch/engadin-skimarathon). Each year, more than 10,000 athletes participate. The race is held in the Canton of Grisons from where Dario Cologna, one of the best cross-country skiers in the World, originates. He has four overall World Cup victories, three Olympic gold medals, one World Championships gold medal, and three Tour de Ski victories in his career (http://www.dariocologna.ch/). In 2007 and 2010, he also won the “Engadin Ski Marathon.”

The aim of this study is now to investigate from where the fastest finishers in “Engadin Ski Marathon” originate. Based upon the knowledge for Kenyan and Ethiopian runners and recent findings for “Norseman Xtreme Triathlon” and “Ironman Hawaii,” we hypothesized that local athletes from Switzerland and especially from the Canton of Grisons would be the fastest.

## 2. Materials and Methods

### 2.1. Ethics Approval

All procedures used in the study were approved by the Institutional Review Board of Kanton St. Gallen, Switzerland, with a waiver of the requirement for informed consent of the participants given the fact that the study involved the analysis of publicly available data.

### 2.2. Methodology

All athletes (*n* = 197,125) who finished the “Engadin Ski Marathon” between 1998 and 2016 were considered. Data were obtained from the publicly available race website of the “Engadin Skin Marathon” at http://www.engadin-skimarathon.ch/.

The “Engadin Ski Marathon” started in 1969, has been a part of the Worldloppet, and is one of the major cross-country skiing events in the Alps. The race is annually held taking place on the second Sunday of March in the upper Engadin valley in Switzerland, Europe, between Maloja and S-chanf. Each year, between 11,000 and 13,000 skiers participate in this race. Since 1998, the total distance covered is 42 km. In that year, the race was extended by 2 km to match the distance of a full marathon and the track was changed slightly in the Stazerwald section, resulting in a more difficult topography, and longer race times are now standard.

### 2.3. Statistical Analysis

All statistical analyses were performed by the statistical package IBM SPSS v. 20.0 (SPSS, Chicago, USA). The figures were created using the software GraphPad Prism v. 7.0 (GraphPad Software, San Diego, USA). Data were presented as mean ± standard deviation. We examined the association of sex with nationality and canton: that is, whether the distribution of sex varied by nationality and canton, chi-square (*χ*^2^) and Cramer's phi (*φ*_*C*_) were used to evaluate the magnitude of association. Men-to-women ratio was calculated for the whole sample and for each nationality and canton. A two-way ANOVA examined the main effects of sex, nationality, and canton (i.e., specific region in Switzerland) and the sex × nationality and sex × canton interaction on race time and age, followed by a Bonferroni post hoc analysis. The magnitude of differences in the ANOVA was evaluated using eta squared (*η*^2^) as trivial (*η*^2^ < 0.01), small (0.01 ≤ *η*^2^ < 0.06), moderate (0.06 ≤ *η*^2^ < 0.14), and large (*η*^2^ ≥ 0.14) [12]. In addition, to study differences in race time and age by sex, nationality, and canton, we used a mixed-effects regression model with finishers as random variable, whereas sex, nationality, and canton were assigned as fixed variables. Also, we examined interaction effects between these fixed variables. Akaike information criterion (AIC) was used to select the final model. Pearson correlation coefficient (*r*) examined the relationship between age and race time. Alpha level was set at 0.05.

## 3. Results

**Figure 1:**
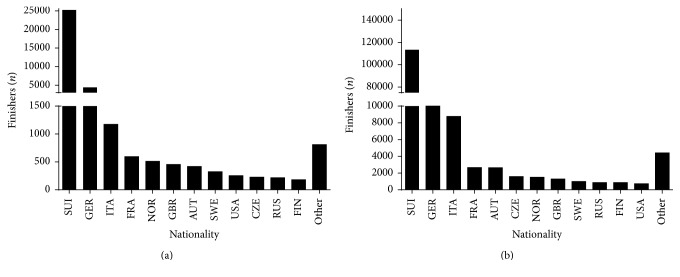
Finishers by nationality in women (a) and men (b). Only nationalities with at least 0.05% finishers of the total number were presented. Nationalities with less than 0.05% were grouped into “others.”

**Figure 2:**
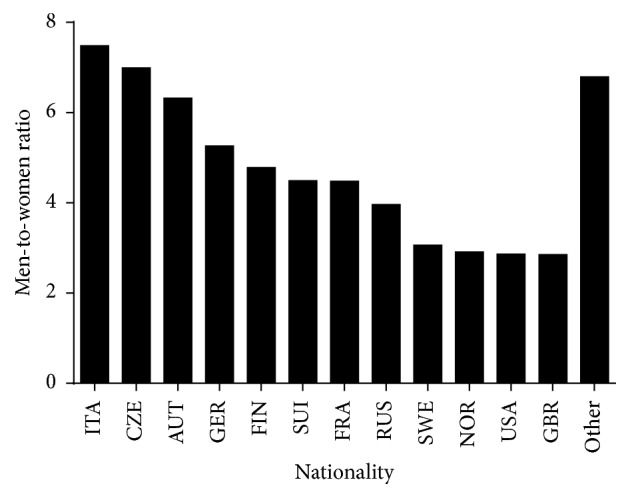
Men-to-women ratio of finishers by nationality. Only nationalities with at least 0.05% finishers of the total number were presented. Nationalities with less than 0.05% were grouped into “others.”

More than two-thirds of finishers (72.5% in women and 69.6% in men) were Swiss and the second, third, and fourth nationalities in prevalence were German, Italian, and French, respectively, in both sexes (Figure 1). The overall men-to-women ratio was 4.68. However, a sex × nationality association was observed (*χ*^2^ = 667.45, *p* < 0.001, Cramer's *φ* = 0.058) with men-to-women ratio ranging from 2.85 (British) to 7.48 (Italian) (Figure 2).

**Figure 3:**
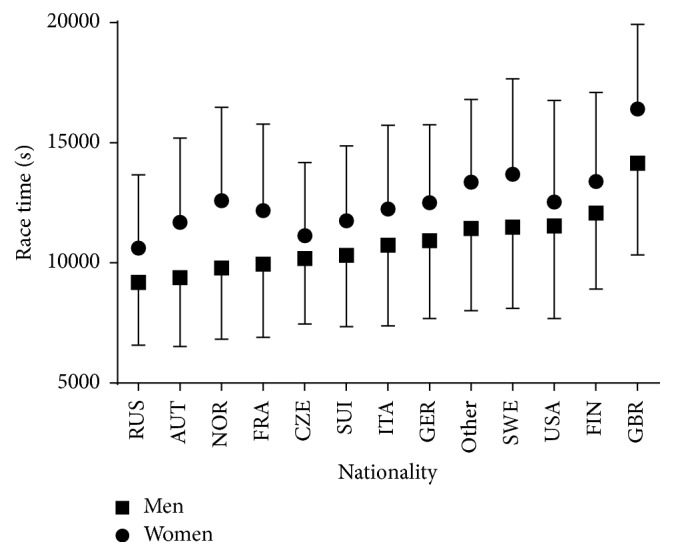
Race time of finishers by nationality. Only nationalities with at least 0.05% finishers of the total number were presented. Nationalities with less than 0.05% were grouped into “others.” Error bars represented standard deviations.

Considering all finishers, a trivial main effect of sex on race time was observed (*p* < 0.001, *η*^2^ = 0.007) with men being faster than women by 12.8% (2:54:31 ± 0:51:31 versus 3:20:02 ± 0:54:33 h:min:s, resp.) (Figure 3, Table 1). A small main effect of nationality on race time was shown (*p* < 0.001, *η*^2^ = 0.020), where the Russians were the fastest and the British were the slowest. A trivial sex × nationality interaction on race time was found (*p* < 0.001, *η*^2^ = 0.001), with the smallest sex difference found in Americans (−8.0%) and the largest in Norwegians (−22.3%).

**Figure 4:**
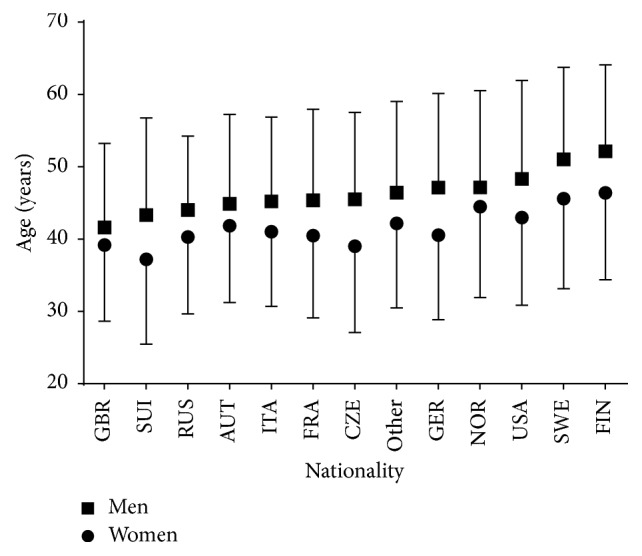
Age of finishers by nationality. Only nationalities with at least 0.05% finishers of the total number were presented. Nationalities with less than 0.05% were grouped into “others.” Error bars represented standard deviations.

With regard to age of all finishers, a trivial main effect of sex was observed (*p* < 0.001, *η*^2^ = 0.003) with women being younger than men by 13.5% (38.3 ± 11.8 versus 44.3 ± 13.3 years, resp.) (Figure 4, Table 2). A small main effect of nationality on age was shown (*p* < 0.001, *η*^2^ = 0.011) with Swiss and British athletes being the youngest and Swedish and Finish athletes the oldest. A trivial sex × nationality interaction on age was found (*p* < 0.001, *η*^2^ = 0.001), with the sex difference in age ranging from −5.7% (Norway) to −14.3% (Czech).

**Figure 5:**
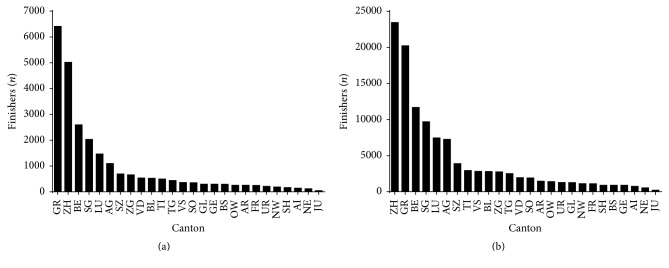
Finishers by canton in women (a) and men (b).

**Figure 6:**
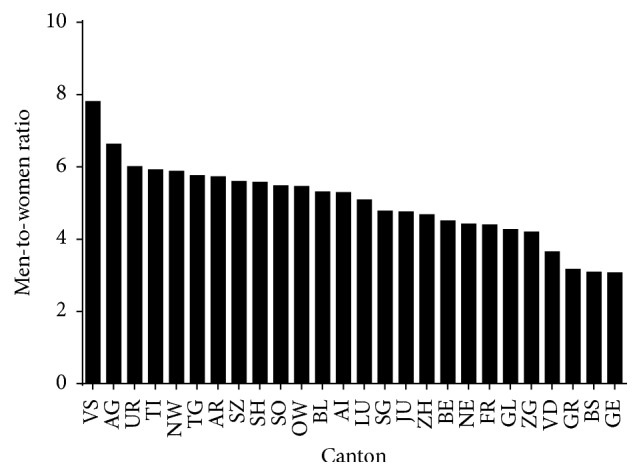
Men-to-women ratio of finishers by canton.

Most of the Swiss finishers (*n* = 138,809) were from Canton of Zurich (20.5%), Grisons (19.2%), and Berne (10.3%). In women (*n* = 25,271), most finishers were from Canton of Grisons (25.4%), Zurich (19.9%), and Berne (10.3%) (Figure 5(a)). In men (*n* = 113,538), most finishers were from Canton of Zurich (20.7%), Grisons (17.8%), and Berne (10.3%) (Figure 5(b)). The overall men-to-women ratio was 4.49. However, a sex × nationality association was observed (*χ*^2^ = 1143.89, *p* < 0.001, Cramer's *φ* = 0.091) with men-to-women ratio ranging from 3.06 (GE) to 7.80 (VS) (Figure 6).

**Figure 7:**
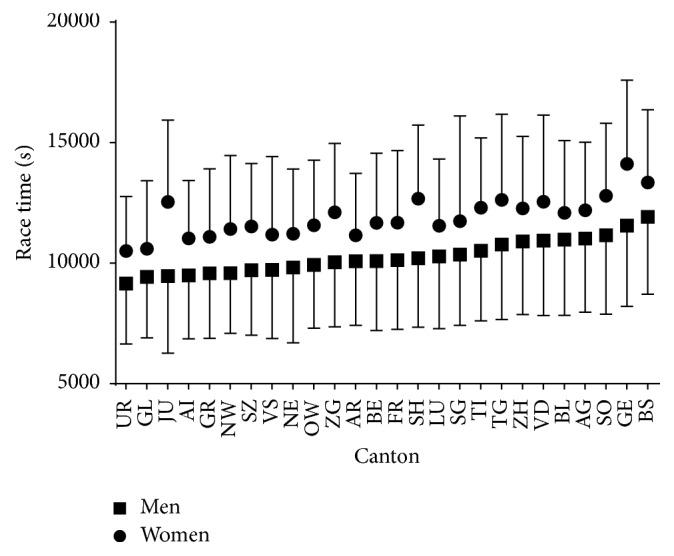
Race time of finishers by canton.

Considering Swiss finishers, a small main effect of sex on race time was observed (*p* < 0.001, *η*^2^ = 0.013) with men being faster than women by 12.3% (2:51:52 ± 0:49:24 versus 3:15:55 ± 0:51:58 h:min:s, resp.) (Figure 7, Table 3). A small main effect of canton on race time was shown (*p* < 0.001, *η*^2^ = 0.023), where finishers from Canton of Uri and Glarus were the fastest and those from Canton of Geneva and Basel the slowest. A trivial sex × canton interaction on race time was found (*p* < 0.001, *η*^2^ = 0.001), with the smallest sex difference found in Canton of Baselland (−9.1%) and the largest in Canton of Geneva (−18.1%).

**Figure 8:**
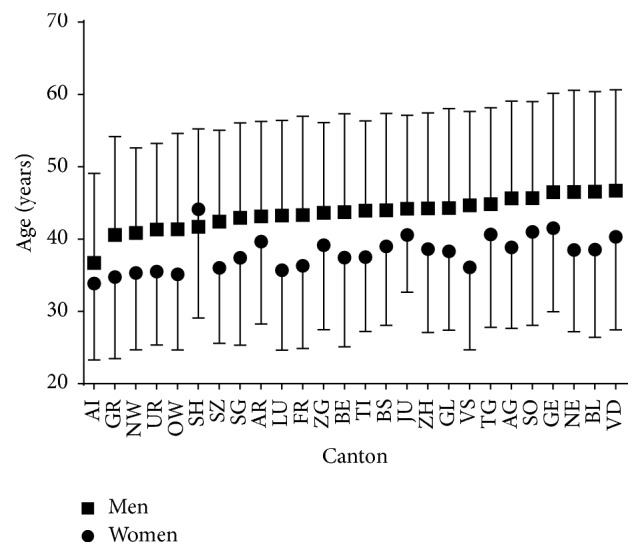
Age of finishers by canton.

With regard to the age of all finishers, a trivial main effect of sex was observed (*p* < 0.001, *η*^2^ = 0.008) with women being younger than men by 14.2% (37.2 ± 11.7 versus 43.3 ± 13.4 years, respectively) (Figure 8, Table 4). A small main effect of canton on age was shown (*p* < 0.001, *η*^2^ = 0.011) with athletes from Canton of Appenzell Innerrhoden being the youngest and athletes from Canton of Geneva the oldest. A trivial sex × canton interaction on age was found (*p* < 0.001, *η*^2^ = 0.001), with the sex difference in age ranging from −7.8% (AI) to −19.2% (VS), whereas there was a canton with older women finishers than men (SH). Small correlations between age and race time were observed in women (*r* = 0.24, *p* < 0.001) and in men (*r* = 0.26, *p* < 0.001).

## 4. Discussion

The main findings of the present study were that (i) more than the two-thirds of finishers were Swiss, (ii) the men-to-women ratio varied by nationality and canton, (iii) there was a trivial sex difference in race time (men were faster) and age (women were younger) which varied by nationality and canton, (iv) Russians were the fastest and the British the slowest, (v) half of Swiss finishers were from Canton of Zurich, Grisons, and Berne, and (vi) Swiss finishers from Canton of Uri and Glarus were the fastest and those from Canton of Geneva and Basel were the slowest.

### 4.1. Most Participants Are Swiss from the Canton of Grisons

We hypothesized that local athletes from Switzerland and especially from the Canton of Grisons would be the fastest. Although most finishers originated from Switzerland, followed by athletes from the surrounding countries Germany, Italy, and France, the fastest finishers originate from Russia. The finding that most finishers were Swiss athletes followed by German, Italian, and French athletes should be attributed to factors related to the popularity of cross-country skiing and the place of the race. Germany, Italy, and France are neighbouring countries to Switzerland. Interestingly, athletes from Austria, which is also near to Switzerland, are not among the most numerous finishers. Most probably, cross-country skiing is not that popular in Austria since alpine skiing is more popular in Austria (http://www.oesv.at/deroesv/index.php). The popularity of a sport might also explain differences in the participation by nationality in the cross-country skiing compared to ultra-marathon running. Particularly, Swiss were less than 50% and Germans were ~40% in the 78 km “Swiss Alpine Marathon” [13]. It is considered that the most powerful form of national performance may be seen in sports [14]. Moreover, the variation of participation by nationality might be due to different attitudes among nationalities towards physical activity [15].

The findings that most Swiss athletes are from Canton of Zurich, Grisons, and Berne is most likely due to the population of the Cantons and the proximity of the race. The Cantons of Zurich and Berne have the most inhabitants of all Cantons in Switzerland (http://www.citypopulation.de/Switzerland-Cities_d.html) and the “Engadin Ski Marathon” is held in the Canton of Grisons.

### 4.2. Russians Are the Fastest in the “Engadin Ski Marathon”

A rather unexpected finding was that athletes from Russia were the fastest since we expected rather athletes from Switzerland to be the fastest. However, cross-country skiers from Russia are among the best in the world. Regarding the data base from FIS, Russia is in third place behind Norway and Sweden when all races and categories are considered for top three positions (https://data.fis-ski.com/cross-country/statistics.html). It has been suggested that the dominance of particular nationalities or ethnic groups in sports is often perceived as evidence of heritage (biological or cultural) [16]. In turn, certain genes have been identified to relate to endurance performance [17]. Another aspect affecting sport performance is doping, for which no accurate rates exist due to its undisclosed practice; however, its prevalence has been estimated as 14–39% in adult elite athletes and has been shown to vary by performance level and nationality [18].

Economic reasons might also explain the dominance of Russian athletes. For example, in some years, the number of foreign visitors could be smaller; there could be also various economic reasons; on websites one may find that starting fees amount to about 1 million and 150 thousands of CHF per year. In some of the followed years, there was world economy crisis. There could be also political reasons; the Russians need travel visa, so they pay and participate at the race when they are highly trained, and really recreational skiers with small economic income participate less.

May be, that among Swiss participant there are more recreational skiers than elite skiers, and therefore, their results are no so excellent. In some Swiss cantons the people may prefer alpine skiing. Scandinavian population may prefer cross-country skiing due to the geography and tradition. In addition to the abovementioned factors, research on other sports has highlighted that psychological factors related to performance might vary by nationality [19, 20]. Also, foreign athletes might perform better than local athletes due to a different interaction with spectators [21].

### 4.3. The Age of the Athletes and Their Origin

Another principal finding of this study was that age of athletes varied by nationality and canton. It might be thought that this variation was related to performance. Actually, a recent study indicated a relationship between age and performance in marathon runners, where the faster runners were younger than the slower [2]. Thus, it might be rationale to assume that fastest nationalities and regions in the present study should be also the youngest. Indeed, athletes from Russia and Austria, which were among the fastest nationalities, had a relatively young age; on the other hand, athletes from Finland, Sweden, and the USA, which were among the slowest, had relatively old age. The way age might impact performance is mostly due to the variation of performance-related physiological characteristics (e.g., aerobic capacity, body composition, and muscle strength) by age; however, age relates to other performance-related factors, such as the choice of equipment [22, 23]. For instance, a recent survey highlighted that 78% of skiers younger than 20 years used helmets in contrast to 53% of those over 60 years [22].

Similarly, athletes from the cantons of AI and UR were relatively fast and young, whereas athletes from the cantons of AG, SO, and GE were relatively slow and old. Moreover, this finding was in agreement with the correlations observed between age and race time. The cantons AI and UR are rather small cantons and located in the mountains whereas the cantons AG, SO, and GE are in the Swiss Central Plateau where snowfall is very rare. So athletes from cantons in the mountains are used to perform skiing and cross-country skiing already early in life in contrast with athletes from cantons in the Swiss Central Plateau.

### 4.4. Limitations, Strength, and Practical Applications

Caution needs to be taken to generalize the findings of the present study to other races, especially in other countries, due to the very large effect of the country of the race on cross-country skiers' participation. On the other hand, strength of the study was the sample size (~200,000 finishers) that allowed comparisons for performance, age, and participation among nationalities. Given the popularity of cross-country skiing, especially in the Nordic countries, the central-European countries, and Russia, the findings interest a large audience. From a theoretical perspective, our findings might help sports scientists to improve their understanding of trends in performance and participation in mega sport events. From a practical perspective, the findings of the present study might help coaches and athletes to optimize their training and competition.

## 5. Conclusions

In the “Engadin Ski Marathon” held between 1998 and 2016, most finishers originated from Switzerland but athletes from Russia were the fastest. Future studies need to investigate other cross-country skiing races in order to find the nationalities and regions of the fastest cross-country skiers and to examine the age of the best cross-country skiing performance.

## Figures and Tables

**Table 1:** Coefficients (*C*) and standard errors (SE) from multivariate regression models for race time by sex and nationality.

Parameter	*C*	SE	*p*
Intercept	10283.55	9.92	<0.001
Nation	92.20	3.07	<0.001
[Sex = women]	1403.10	23.50	<0.001
[Sex = men]	0^*∗*^	0^*∗*^	
[Sex = women] × nationality	63.34	7.23	<0.001
[Sex = men] × nationality	0^*∗*^	0^*∗*^	

^*∗*^This parameter was set to zero because it was redundant.

**Table 2:** Coefficients (*C*) and standard errors (SE) from multivariate regression models for age by sex and nationality.

Parameter	*C*	SE	*p*
Intercept	43.54	0.04	<0.001
Nation	0.36	0.01	<0.001
[Sex = women]	−6.44	0.10	<0.001
[Sex = men]	0^*∗*^	0^*∗*^	
[Sex = Women] × nationality	0.23	0.03	<0.001
[Sex = men] × nationality	0^*∗*^	0^*∗*^	

^*∗*^This parameter was set to zero because it was redundant.

**Table 3:** Coefficients (*C*) and standard errors (SE) from multivariate regression models for race time by sex and canton.

Parameter	*C*	SE	*p*
Intercept	9496.96	17.76	<0.001
Canton	66.67	1.26	<0.001
[Sex = women]	1522.96	38.87	<0.001
[Sex = men]	0^*∗*^	0^*∗*^	
[Sex = women] × Canton	−1.74	2.87	0.544
[Sex = men] × Canton	0^*∗*^	0^*∗*^	

^*∗*^This parameter was set to zero because it was redundant.

**Table 4:** Coefficients (*C*) and standard errors (SE) from multivariate regression models for age by sex and canton.

Parameter	*C*	SE	*p*
Intercept	40.29	0.08	<0.001
Canton	0.25	0.01	<0.001
[Sex = women]	−5.68	0.17	<0.001
[Sex = men]	0^*∗*^	0^*∗*^	
[Sex = women] × Canton	−0.02	0.01	0.094
[Sex = men] × Canton	0^*∗*^	0^*∗*^	

^*∗*^This parameter was set to zero because it was redundant.

## References

[1] Marc A., Sedeaud A., Schipman J. (2017). Geographic enrolment of the top 100 in athletics running events from 1996 to 2012. *Journal of Sports Medicine and Physical Fitness*.

[2] Nikolaidis P. T., Onywera V. O., Knechtle B. (2017). Running performance, nationality, sex, and age in the 10-km, half-marathon, marathon, and the 100-km ultramarathon IAAF 1999–2015. *Journal of Strength and Conditioning Research*.

[3] Onywera V. O., Scott R. A., Boit M. K., Pitsiladis Y. P. (2006). Demographic characteristics of elite Kenyan endurance runners. *Journal of Sports Sciences*.

[4] Scott R. A., Georgiades E., Wilson R. H., Goodwin W. H., Wolde B., Pitsiladis Y. P. (2003). Demographic characteristics of elite Ethiopian endurance runners. *Medicine and Science in Sports and Exercise*.

[5] Rüst C. A., Knechtle B., Knechtle P., Lepers R., Rosemann T., Onywera V. (2014). European athletes dominate performances in double iron ultra-triathlons—a retrospective data analysis from 1985 to 2010. *European Journal of Sport Science*.

[6] Rüst C. A., Bragazzi N. L., Signori A., Stiefel M., Rosemann T., Knechtle B. (2015). Nation related participation and performance trends in ‘Norseman Xtreme Triathlon’ from 2006 to 2014. *SpringerPlus*.

[7] Dähler P., Rüst C. A., Rosemann T., Lepers R., Knechtle B. (2014). Nation related participation and performance trends in 'Ironman Hawaii' from 1985 to 2012. *BMC Sports Science, Medicine and Rehabilitation*.

[8] Knechtle B., Rüst C., Rosemann T. (2013). The aspect of nationality in participation and performance in ultra-marathon running—a comparison between 'Badwater' and 'Spartathlon'. *OA Sports Medicine*.

[9] Knechtle B., Rosemann T., Rüst C. A. (2014). Participation and performance trends by nationality in the 'english channel swim' from 1875 to 2013. *BMC Sports Science, Medicine and Rehabilitation*.

[10] Rüst C. A., Knechtle B., Rosemann T. (2013). The relationship between nationality and performance in successful attempts to swim across the ‘English Channel’—a retrospective data analysis from 1875 to 2012. *Medicina Sportiva*.

[11] Carlsson T., Carlsson M., Assarsson H. (2016). The influence of sex, age, and race experience on pacing profiles during the 90 km Vasaloppet ski race. *Open Access Journal of Sports Medicine*.

[12] Cohen J. (1988). *Statistical Power Analysis for The Behavioral Sciences*.

[13] Eichenberger E., Knechtle B., Rüst C. A., Lepers R., Rosemann T., Onywera V. O. (2012). The aspect of nationality and performance in a mountain ultra-maratho-the 'Swiss Alpine Marathon'. *Journal of Human Sport and Exercise*.

[14] Bairner A. (2015). Assessing the sociology of sport: on national identity and nationalism. *International Review for the Sociology of Sport*.

[15] Ntoumanis N., Biddle S. J. H. (1999). Affect and achievement goals in physical activity: a meta-analysis. *Scandinavian Journal of Medicine and Science in Sports*.

[16] Rupert J. L. (2003). The search for genotypes that underlie human performance phenotypes. *Comparative Biochemistry and Physiology—A Molecular and Integrative Physiology*.

[17] Collins M., Xenophontos S. L., Cariolou M. A. (2004). The ACE gene and endurance performance during the South African Ironman Triathlons. *Medicine and Science in Sports and Exercise*.

[18] de Hon O., Kuipers H., van Bottenburg M. (2014). Prevalence of doping use in elite sports: a review of numbers and methods. *Sports Medicine*.

[19] Golby J., Sheard M., Lavallee D. (2003). A cognitive-behavioural analysis of mental toughness in national rugby league football teams. *Perceptual and Motor Skills*.

[20] Sheard M. (2009). A cross-national analysis of mental toughness and hardiness in elite university rugby league teams. *Perceptual and Motor Skills*.

[21] Poulter D. R. (2009). Home advantage and player nationality in international club football. *Journal of Sports Sciences*.

[22] Ruedl G., Sommersacher R., Woldrich T. (2010). Who is wearing a ski helmet? Helmet use on austrian ski slopes depending on-various factors. *Sportverletzung-Sportschaden*.

[23] Ruedl G., Pocecco E., Kopp M. (2015). Factors associated with the use of protective gear among adults during recreational sledging. *Sportverletzung-Sportschaden*.

